# Effects of Occlusal Contact on Maxillary Alveolar Bone Morphology in Patients with and without Anterior Open Bite: A Cross-Sectional Study

**DOI:** 10.3390/jcm13113061

**Published:** 2024-05-23

**Authors:** Chiyo Shimizu-Tomoda, Yuji Ishida, Aiko Ishizaki-Terauchi, Yukari Mizoguchi, Shuji Oishi, Takashi Ono

**Affiliations:** Department of Orthodontic Science, Graduate School of Medical and Dental Sciences, Tokyo Medical and Dental University (TMDU), 1-5-45 Yushima, Bunkyo-ku, Tokyo 113-8549, Japan; shimizu.chiyo@tmd.ac.jp (C.S.-T.);

**Keywords:** anterior open bite, maxillary alveolar bone, occlusal contacts, bite force, cross-sectional study

## Abstract

**Background/Objectives:** Anterior open bite (AOB) is characterized by the absence of occlusal contact between the maxillary and mandibular anterior teeth, while the posterior teeth are in contact when occluded. Here, we aimed to clarify the difference in maxillary alveolar bone morphology in adult patients with and without AOB. **Methods:** This cross-sectional study was conducted on 50 adults aged 18–39 years: 25 patients without AOB (control group; 13 males and 12 females; age: mean ± standard deviation [SD], 22.2 ± 4.5 years) and 25 patients with AOB (9 males and 16 females; age: 24.2 ± 6.4 years). Using cone-beam computed tomography images, the height of the maxillary alveolar bone crest in the anterior and posterior teeth and thickness of the alveolar cortical bone on the labial and palatal sides were measured and compared between the two groups. An independent *t*-test and Pearson’s correlation analysis were used to examine statistical significance (*p* < 0.05). **Results:** The AOB group showed a significantly longer (*p* = 0.016) posterior alveolar crest and thinner cortical bone on the buccal (*p* < 0.001) and lingual (*p* = 0.009) sides of the anterior region and the buccal (*p* = 0.006) sides of the posterior region than the control group did. Moreover, a significant negative correlation (*p* = 0.046; r = −0.403) was observed between bite force and cortical bone thickness on the buccal side of the posterior region in the AOB group. **Conclusions:** It is suggested that the absence of occlusal contact in the anterior area influences the alveolar bone morphology of the maxilla.

## 1. Introduction

Anterior open bite (AOB) is an occlusal condition characterized by the absence of vertical overlap between the maxillary and mandibular anterior teeth when the posterior teeth are in occlusion [1]. The incidence of AOB ranges from 1.5% to 11% and varies between races and with dental age [2]. In the mixed dentition, the prevalence of AOB can reach up to 18.5%, decreasing with age [3]. AOB leads to masticatory disorders, such as inability to bite with the anterior teeth, excessive load concentrated on the molars and/or temporomandibular joints, aesthetic disorders such as lack of overlap in the anterior teeth, speech problems, psychological damage, and breathing disorders [4,5]. In growing adolescents, AOB has adverse effects on maxillofacial growth [6]. AOB is derived from the interactions of many factors, including abnormal habits such as thumb sucking, tongue protrusion upon swallowing, mouth breathing, and hereditary factors such as facial growth pattern. Hence, the etiology of AOB remains unclear [7].

The aesthetic and functional problems associated with AOB can be improved orthodontically by using a palatal tongue crib, bonded lingual spurs, orofacial myofunctional therapy, extrusion of the anterior teeth, and/or deformity correction with surgical intervention. Temporary anchorage devices (TADs) have enabled AOB treatment via mandibular autorotation with the intrusion of the posterior teeth. Practitioner preferences, patient demographics, and patients’ dentofacial characteristics influence the treatment options [8]. While fixed appliances and elastics are the most common treatments for adults, one-third of practitioners use aligners, and approximately 10% recommend TADs or orthognathic surgery [9].

In patients with AOB, mechanical stimulation during mastication is insufficient in the anterior teeth of both jaws, which may modify the alveolar bone morphology. Animal experiments have shown that reducing masticatory stimuli leads to a decrease in alveolar bone volume in rats [10]. An increased masticatory and/or bite force promotes bone formation in the masseter muscle attachment site, resulting in robust mandibular growth in mice [11]. In contrast, a reduction in occlusal loading induces a simultaneous response in the narrower alveolar process and periodontal ligament in the rat maxilla [12]. Moreover, it is suggested that changes in mastication markedly affect the mandibular condylar cartilage growth and mandibular morphology [13], and a functional adaptive response by the mandible to mechanical stress resulting from mastication occurs in the muscle insertion area and mandibular alveolar bone in the molar region [14]. Furthermore, morphometric studies in animals have suggested that the alveolar bone is influenced by masticatory function [15,16,17]. In humans, the maximum bite force had a selective influence on alveolar thickness and shape [18], and a positive relationship was observed between masticatory functions, as determined by masseter thickness and the number of occluding posterior teeth, as well as alveolar bone mass and thickness [19]. Notably, bone growth mass and direction (i.e., height and anteroposterior relationship) can change with some influence from the functional stimuli, and bite forces are expected to be involved in alveolar bone morphology. However, no studies have investigated the effects of occlusal contacts on maxillary alveolar bone morphology in humans.

This study aimed to elucidate the relationship between the condition of occlusal contact and maxillary alveolar bone morphology by comparing subjects with and without AOB. We investigated whether a difference existed in the alveolar maxillary bone morphologies between the AOB and control groups, challenging the null hypothesis.

## 2. Materials and Methods

### 2.1. Samples

This cross-sectional study was approved by the Institution Ethical Committee of Tokyo Medical and Dental University (TMDU) (approval numbers: No. D2015-609). This study included pre-treatment cone-beam computed tomography (CBCT) data from 50 orthodontic patients (22 males and 28 females) who underwent CBCT examination for orthodontic diagnosis due to impaction of the third molars at the orthodontic clinic between April 2017 and November 2019.

The inclusion criteria were as follows: (1) patients aged 18–39 years; (2) complete root formation of the permanent teeth; and (3) no history of previous orthodontic treatment. The exclusion criteria were as follows: (1) history of trauma to the maxillary anterior teeth; (2) radiographic signs of periodontitis [20] or periapical lesions; (3) history of orthodontic and/or prosthodontic treatment; (4) abnormal number of teeth, such as congenitally missing or supernumerary teeth; (5) medication related to bone metabolism; and (6) presence of craniofacial anomalies. The sample size was determined using G*power 3.1.9.6 (Franz Faul Universitat Kiel, Kiel, Germany), as briefly described below. Based on previous studies on alveolar bone morphologies [21,22], the power was set at 0.80 with a two-sided significance level of 0.05, and the effect size was set at 0.80. Based on power analysis, 26 patients from each group were required. This sample size was not significantly different from those used in previous studies [23,24]. Therefore, we randomly collected 26 CBCT data for both groups from the archived database: the control group (13 males and 12 females; mean age: 22.2 ± 4.5 years) with a positive overbite (overbite ≥ 0 mm, with occlusal contact in the anterior region), and the AOB group (9 males and 16 females; mean age: 24.2 ± 6.4 years) with AOB (overbite < 0 mm, without occlusal contact in the anterior region). One patient in each group was removed following the exclusion criteria. Informed consent was obtained from all patients. CBCT images (Finecube; YOSHIDA Dental Manufacturing, Tokyo, Japan) (slice thickness: 0.147 mm; fields of view: 81 × 74 mm; and voxel size: 0.146 mm) were acquired using the normal mode: 16.8 s, 4.10 mGy, 90 kV, and 4 mA. Images were captured with the Frankfort horizontal plane parallel to the floor and saved as Digital Imaging and Communication in Medicine (DICOM) files. CT images were reconstructed and analyzed using three-dimensional (3D) image reconstruction software (OsiriX MD, version 8.0.2, Pixmeo, Geneva, Switzerland).

### 2.2. Measurements

**Figure 1:**
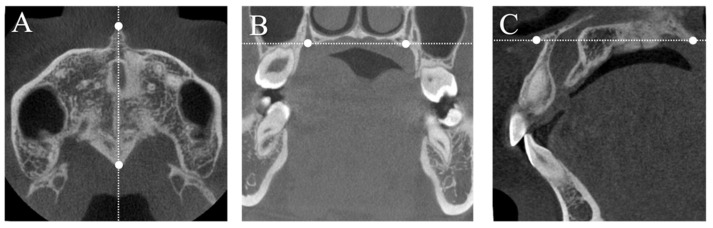
Landmarks, reference lines, and planes drawn on cone-beam computed tomography images. (**A**) The axial plane with reference to the mid-palatal plane through ANS and PNS; (**B**) the coronal plane perpendicular to the axial plane and passing through the right and left greater palatine foramina; (**C**) the sagittal plane perpendicular to both the axial and coronal planes and passing through the mid-palatal suture. Abbreviations: ANS, anterior nasal spine; PNS, posterior nasal spine.

**Figure 2:**
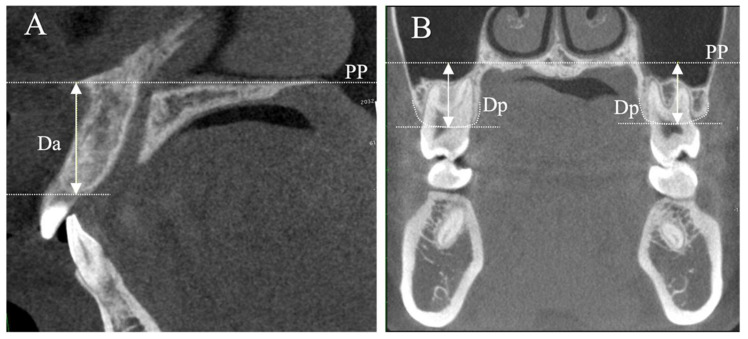
Linear measurements. (**A**) sagittal view; (**B**) coronal view. Abbreviations: PP, the palatal plane; Da, distance between the anterior alveolar crest and the PP on the sagittal image; Dp, average distance between the posterior alveolar crest and PP in the maxillary first molars, calculated as the mean of the left and right sides on the coronal image.

Information regarding sex and age was obtained from clinical records. Using dental casts, the overbite was measured as the distance (in mm) between the incisal edges of the maxillary and mandibular incisors, perpendicular to the occlusal plane. Lateral cephalometric radiographs (Hyper-X, ASAHI., Tokyo, Japan) were used to measure the cephalometric variables [25,26]. The skeletal relationship was evaluated with the angle formed by the nasion-point A line and nasion-point B line (ANB angle); the facial pattern with the angle formed by the Frankfort horizontal (FH) plane and mandibular plane (MPA); and the angle formed by the sella-nasion line and gonion-gnathion line (SN-GoGn angle). To ensure consistent adjustment of the CBCT images, the following reference planes were used: (1) the axial plane with reference to the mid-palatal plane through the anterior nasal spine (ANS) and posterior nasal spine (PNS) (Figure 1A); (2) the coronal plane perpendicular to the axial plane and passing through the right and left greater palatine foramina (Figure 1B); and (3) the sagittal plane perpendicular to both the axial and coronal planes and passing through the mid-palatal suture (Figure 1C). Before the measurements, the 3D images were calibrated, and the three planes (axial, coronal, and sagittal) were defined in each image. Linear measurements were performed in the sagittal and coronal planes (Figure 2).

Da: distance between the anterior alveolar crest and the palatal plane (PP).

Dp: average distance between the posterior alveolar crest and PP in the maxillary first molars, calculated as the mean of the left and right sides on the coronal image.

**Figure 3:**
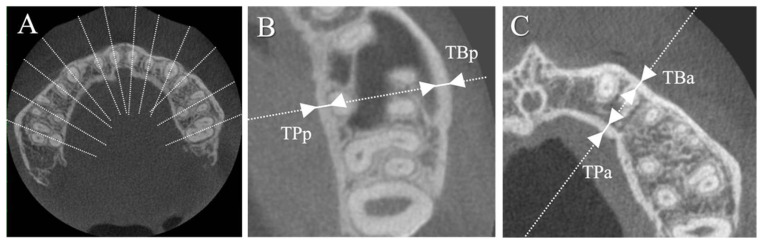
Linear measurements on the horizontal image. (**A**) Buccolingual bisectors for each tooth are shown in the horizontal image. (**B**) The thickness (mm) of the buccal (TB) and palatal (TP) cortical bone is measured from a magnified image of the posterior area and (**C**) the anterior area.

In all the maxillary teeth, the buccal and palatal cortical bone thicknesses (TB, thickness of buccal cortical bone; TP, thickness of palatal cortical bone) were measured on the axial plane at the apical level of each tooth. For multiple roots, the root with the longest length was used for the measurement. The cortical bone thickness values of the anterior region (i.e., TBa and TPa) were calculated from the average of the measurements of all six anterior teeth from the right to the left canine. Cortical bone thickness values of the posterior region (i.e., TBp and TPp) were calculated from the average of the values of all molars (Figure 3). Premolars were excluded from group comparisons because some were in occlusal contact and others were not.

Finally, for bite force measurement, we used the Dental Prescale 50H (GC Corp., Tokyo, Japan), a dedicated scanner (GT-X830, EPSON, Tokyo, Japan), and analysis software (Bite Force Analyzer, version 2.2.0, GC Corp., Tokyo, Japan). In the experiment, a prescale of an appropriate size was inserted into the oral cavity so that the entire dentition fit in the film. The subject was instructed to perform maximal clenching for approximately 3 s in the intercuspal position. Given the established reliability achieved through pre-experimental practice, bite force was measured once after the subject was instructed on and had practiced the method for performing maximum clenching before the examination. The maximum bite forces were calculated from the pressure and area at the contact points using the software (Bite Force Analyzer, GC, Tokyo, Japan) [27,28].

### 2.3. Statistical Analyses

All statistical analyses were performed using IBM SPSS Statistics version 26.0 (IBM, Armonk, NY, USA). The Shapiro–Wilk test results suggest that the data were normally distributed in all groups; therefore, parametric tests were used to perform within and between-group comparisons. An independent t-test was performed to examine differences between the control and AOB groups regarding age, overbite, ANB, Frankfort (FH) mandibular plane angle, SN-GoGn angle, angle formed by the maxillary incisor axis (U1 to FH) and FH plane, bite force, and the thickness of the cortical bone and alveolar crest height. Chi-square tests (χ^2^) were used to compare the sex proportion. Demographic and cephalometric measurements were presented as mean values and standard deviations (SD). Pearson’s correlation analysis was performed to evaluate the correlations between the cortical bone thickness and bite force. Statistical significance was set at *p* < 0.05.

## 3. Results

All the measurements were performed by a single examiner. After a 2-week interval, 25 samples were randomly selected and remeasured to check the intra-examiner reliability. The intraclass correlation coefficient (ICC) values for all the measurements ranged between 0.977 and 0.993, indicating high reliability. The ICCs between the first and second measurements for Da, Dp, TB, and TP were >0.9 [29]. To ensure the reliability of the measurements for statistical analysis, the same examiner repeated the measurements after an interval of 2 weeks. The method error was determined using Dahlberg’s formula [30,31]. The method error of the cephalometric analysis ranged from 0.142 mm to 0.194 mm for the linear measurements and from 0.161° to 0.674° for the angular measurements. The method error of the 3D CT analysis ranged from 0.03 mm to 0.35 mm. The reliability of the data was evaluated by calculating the intraclass correlation coefficient (ICC). The ICCs for the cephalometric and CT measurements ranged from 0.96 to 0.99 and from 0.77 to 0.99, respectively.

Comparisons of sex, age, overbite, and cephalometric analysis between the control and AOB groups are shown in Table 1. For facial pattern analysis, no significant differences were observed in ANB, MPA, and SN-GoGn between the two groups. In other words, there were no significant differences observed in any variables except for overbite.

Descriptive statistics of all the measurements of cortical bone thickness and alveolar bone crest height in the control and AOB groups are shown in Table 2. Dp, TBa, TPa, and TBp showed significant differences between the two groups. The Dp in the AOB group (15.09 ± 1.38 mm) was significantly larger (*p* = 0.016) than that in the control group (14.12 ± 1.35 mm). In contrast, the TBa in the AOB group (1.19 ± 0.13 mm) was significantly smaller (*p* < 0.001) than that in the control group (1.41 ± 0.17 mm). The TPa (1.25 ± 0.16 mm) and TBp (1.25 ± 0.24 mm) regions in the AOB group were significantly smaller (*p* = 0.009 and *p* = 0.006, respectively) than those (1.44 ± 0.32 mm and 1.44 ± 0.23 mm, respectively) in the control group. The TPp in the AOB group was thinner than that in the control group, although the difference was not statistically significant.

**Figure 4:**
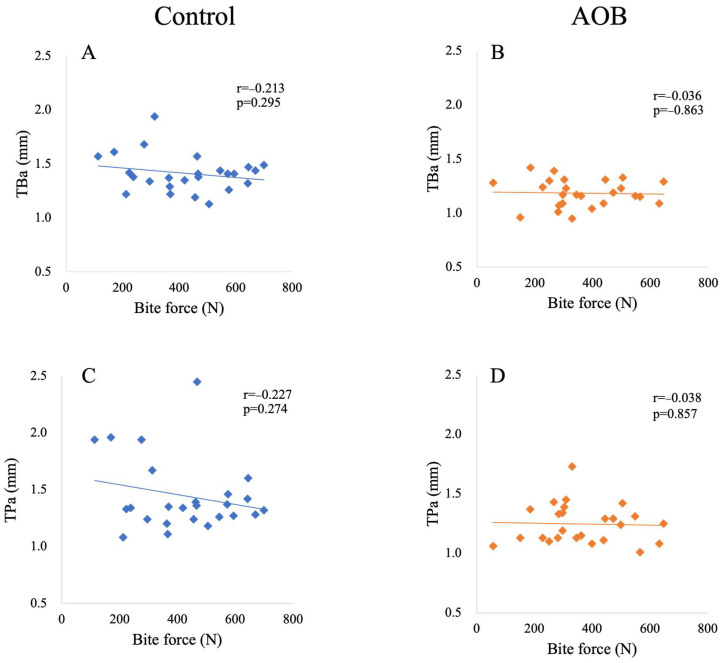
Relationship between bite force and cortical bone thickness in the anterior region in participants with normal bite and anterior open bite. Each plot indicates the bite force of a subject (*X*-axis) (N) and cortical bone thickness (*Y*-axis) (mm). (**A**) Relationship between bite force and cortical bone thickness of the buccal side in participants with normal bite; (**B**) relationship between bite force and cortical bone thickness of the buccal side in participants with anterior open bite; (**C**) relationship between bite force and cortical bone thickness of the palatal side in participants with normal bite; (**D**) relationship between bite force and cortical bone thickness of the palatal side in participants with anterior open bite. Abbreviations: AOB, participants with anterior open bite; TBa, cortical bone thickness of the buccal side of the anterior region; TPa, cortical bone thickness of the palatal side of the anterior region; r, correlation coefficient; *p*, probability.

**Figure 5:**
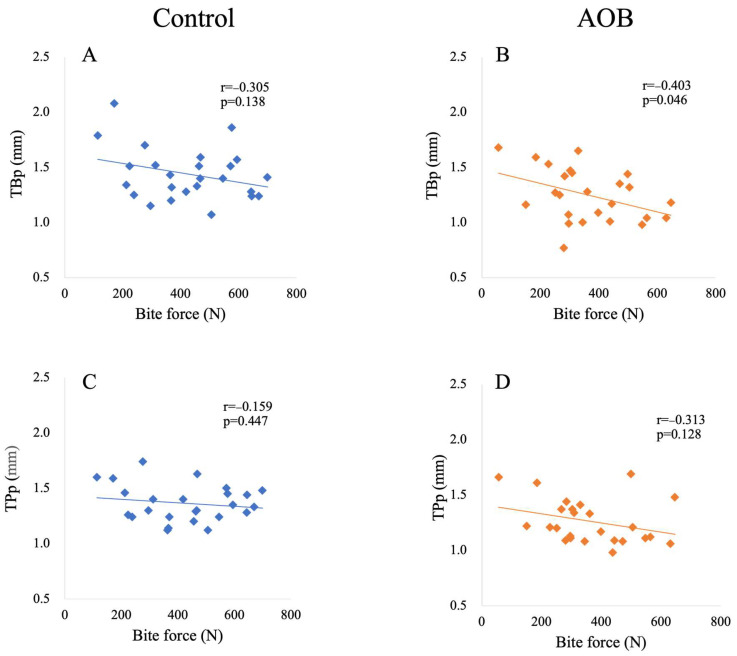
Relationship between bite force and cortical bone thickness in the posterior region in participants with normal bite and anterior open bite. Each plot indicates the bite force of a subject (*X*-axis) (N) and cortical bone thickness (*Y*-axis) (mm). (**A**) Relationship between bite force and cortical bone thickness of the buccal side in participants with normal bite; (**B**) relationship between bite force and cortical bone thickness of the buccal side in participants with anterior open bite; (**C**) relationship between bite force and cortical bone thickness of the palatal side in participants with normal bite; (**D**) relationship between bite force and cortical bone thickness of the palatal side in participants with anterior open bite. Abbreviations: AOB, participants with anterior open bite; TBp, cortical bone thickness of the buccal side of the posterior region; TPp, cortical bone thickness of the palatal side of the posterior region; r, correlation coefficient; *p*, probability.

Table 3 presents the relationship between bite force and cortical bone thickness in both the control and AOB groups. In the anterior region, no significant correlation was found between bite force and the cortical bone thickness on the buccal (i.e., TBa) and palatal (i.e., TPa) sides in either the control or AOB groups (Figure 4). However, in the posterior region, a significant correlation (*p* = 0.046; r = −0.403) was observed between bite force and cortical bone thickness on the buccal side (i.e., TBp) (Figure 5).

## 4. Discussion

This cross-sectional study aimed to elucidate the relationship between the anterior occlusal contacts and the morphology of the maxillary alveolar bone, which lacks attachment sites for masticatory muscles, in patients with or without masticatory-related stimuli. Numerous reports have explored the connection between function and morphology. For instance, a study on the direction of craniofacial morphogenesis [32] found that a decrease in masticatory stimulation leads to a reduction in the surrounding alveolar bone volume of the mandible [10], while increased masticatory and bite forces promotes bone formation at the masseter muscle attachment area, resulting in robust growth of the maxilla and mandible in animal experiments [11]. However, few clinical studies have investigated the relationship between occlusal stimulation and the jawbone. To the best of our knowledge, this is the first study to date that examines the correlation between occlusal contacts, such as the presence or absence of AOB, bite force, and alveolar bone morphology, including cortical bone thickness and height of the maxillary alveolar bone, using CBCT imaging.

In conventional orthodontics, diagnosis and treatment planning are generally performed using two-dimensional (2D) radiographs including cephalometric, panoramic, and periapical radiographs. However, these 2D analyses have several limitations, such as poor representations of bone thickness and width and reproduction errors due to low reproducibility. Moreover, periapical radiographs cause distortion and structural overlap [33]. Furthermore, in panoramic radiographs, the vertical magnification often varies owing to the orientation of the patients, resulting in the distortion of images, especially in the vertical dimension. Therefore, precise analysis of 3D structures can pose challenges when relying solely on conventional 2D images [34,35,36]. In contrast, CBCT can provide undistorted and high-resolution 3D views of hard tissues [37]. Therefore, CBCT imaging is a suitable modality for the detailed investigation and analysis of alveolar bone morphology [38]. Our results demonstrate that the cortical bone width was significantly thinner in the AOB group than in the control group, on both sides of the maxillary alveolar bone in the anterior tooth region on which occlusal stimuli were not loaded during mastication in the AOB group. In the posterior region, the cortical bone width was significantly thinner in the AOB group than in the control group on the buccal side only, whereas no significant difference was observed on the palatal side. The results also show that the alveolar crest height was predominantly greater in the posterior region of the AOB group than in the control group. Most previous studies are based on cephalometric measurements, which evaluate the height of the central incisors and molars concerning the mandibular plane and PP [39]. These previous studies have reported increased alveolar bone height of the molars in AOB groups, which is consistent with the results of this study [40]. Kucera et al. [41] have reported that alveolar bone height for the anterior teeth was significantly higher in the skeletal open bite group than in the control group; this is inconsistent with our results. The cephalometric radiograph does not accurately reflect bone morphology, such as alveolar bone thickness and height, which should be taken into account [42]. In 3D imaging studies, Tang et al. [24] reported that patients with AOB exhibited a larger alveolar bone height in the molar region than patients without AOB did, which is consistent with our results. Furthermore, the bucco–palatal alveolar bone width in the AOB group was smaller than that in the control group in the maxillary molar region. Additionally, we observed that the buccal cortical bone was significantly thinner in the AOB group than in the control group, which is consistent with the findings of Tang et al. [24].

This study focused on clarifying the relationship between the occlusal condition and morphology of the maxillary alveolar bone based on the assumption that a relationship exists with the direction of bone remodeling. Generally, bone grows and develops through apposition at the proximal and distal ends. Bone remodeling occurs at various points and ends. Previous studies have argued that bone remodeling involves appositions (formation) and resorptions (removals) that occur concurrently, driving relative movement between bone elements and ultimately leading to bone transformation [43]. When individual bones are simultaneously remodeled at each site, they are all remodeled simultaneously as a single functional complex, as in the nasal–maxillary complex [44,45]. This study shows that cortical bone thinning on the labial side in the anterior to posterior regions and an increased height of the alveolar crest in the molar area within the AOB group aligns with the observations made by Enlow et al. [43,44]. According to Enlow’s law, the maxillary complex grows in an antero–inferior direction during growth periods, with bone resorption occurring concomitantly with bone apposition. Therefore, there must be a positive shift to bone resorption on the buccal side, whereas on the palatal side there is a shift towards bone apposition. This phenomenon helps explain why the labial cortical bone exhibited significant thinning, whereas the palatal cortical bone did not demonstrate the same trend, particularly in the posterior region of the AOB group. In other words, the absence of mechanical stimulation on the anterior teeth in the AOB group suggests that the nasal–maxillary complex likely experiences pronounced forward and downward movement compared to that in the control group. In contrast, the cortical bone of the anterior teeth on the palatal side was significantly thinner in the AOB group than in the control group, whereas no significant difference was observed in the cortical bone thickness of the posterior teeth. According to Dean et al. [46], bone remodeling of the palate is slightly different from that of the labial side; bone resorption and apposition occur on the nasal and palatal sides, respectively. Additionally, attention should be directed towards the fact that the measurement site on the palatal side of the anterior teeth in this study was positioned anterior to the incisor canal. Previous studies have indicated distinct developmental origins for the human palate, with the anterior part resembling the nasal side and the posterior part representing the palatal side beyond the incisor canal [47]. Considering this perspective, if we view the anterior part of the palate measured in this study as bone remodeled similarly to that on the nasal cavity side, the finding of significantly thinner cortical bone in the AOB group than in the control group is not contradictory As increased molar height is a common feature of skeletal open bite, various opinions exist on whether the height of the maxillary and mandibular molars increases [41,48,49]. Isaacson et al. [49] concluded that the height of the maxillary posterior alveolar process is the most important factor in determining the height of the mandibular plane growth pattern. In several studies, an increase in the maxillary molar height only was found in open bite malocclusions [50,51]. In this study, the height of the alveolar crest was significantly increased only in the posterior region in the AOB group, compared to that in the control group, with no skeletal difference. Urzal et al. [52] suggested that abnormal oral habits, such as finger sucking and abnormal tongue swallowing, are risk factors for AOB development. Therefore, the soft tissue mediation of oral habits suppressed anterior downward bone growth in the maxillary anterior region in the AOB group, resulting in no significant difference in the alveolar crest height between the AOB and control groups [53].

Many studies on the alveolar compensatory mechanism and alveolar bone morphology of skeletal open bite have produced controversial debates [39,40,41]. Kucera et al. [41] reported that skeletal open bite is compensated by a large anterior height and incisor elongation and inclination. Arriola-Guillén et al. [54] found that a skeletal class II open bite group showed greater maxillary incisor height than did class I controls. Other studies have reported lower incisor heights in patients with AOB than in controls [55,56]. Our results focused on the alveolar crest height, and the alveolar bone height in the anterior part tended to be lower in the AOB group than in the control groups, with no significant difference. Finally, few previous studies on CBCT have focused on the relationship between bite force and alveolar bone morphology [57,58,59]. In this study, we analyzed the correlation between bite force and cortical bone thickness and found a significant negative correlation between buccal molar cortical bone thickness and bite force in the AOB group, and a negative correlation between the cortical bone thickness and bite force in the control group without significance, whereas no correlation was observed in the anterior region in the AOB group. This is contrary to previous findings, which stipulate that stimulation of the jawbone by mastication promotes bone formation [11,18], resulting in robust bone growth. However, these effects of bone formation were especially found around muscular attachment areas with direct muscular stimuli, whereas this study focused on the alveolar bone at the root apex level apart from muscular attachment areas. Interestingly, our results demonstrated no correlation in the anterior part of patients with AOB without masticatory stimuli, suggesting that bite force influences the thickness of the maxillary alveolar bone, particularly in the posterior part of patients with AOB. Further studies are necessary to elucidate the relationship between masticatory stimuli and cortical and cancellous bone formation in the future.

This study has several limitations. First, it is based on pre-treatment cross-sectional data at only one time point. Longitudinal studies are necessary to investigate the correlation between bite force and bone modeling more thoroughly. Elucidating the relationship between occlusal stimulation and maxillofacial bone growth will provide insights into the optimal timing of orthodontic treatment for children with an open bite, determining whether treatment should be administered before or after an adolescent growth spurt. Second, this study focused on maxillary bone changes; therefore, a detailed investigation of the mandibular alveolar bone is required to clarify the relationship between mechanical stimulation and mandibular alveolar bone morphology. Third, when selecting samples, skeletal conditions were not considered, and patients were not recruited under certain conditions regarding anatomical variations; thus, there is a possibility of anatomical variations. Fourth, further studies are required to determine the amount of change between the anatomical features before and after orthodontic treatment that acquired a stable and functional occlusion. Clinicians must consider that stresses along supporting tissues can change via the bone remodeling response associated with ongoing tooth movement and changes in the force system.

## 5. Conclusions

From the CBCT analyses, the AOB group with an absence of the occlusal contact in the anterior regions showed a larger posterior alveolar crest height and thinner cortical bone on the buccal and lingual sides of the anterior teeth and on the buccal sides of the posterior teeth in comparison with the control group. Furthermore, in the AOB group, a moderate negative correlation was observed between bite force and cortical bone thickness on the buccal side of the posterior teeth. Hence, anterior occlusal contacts influence the morphology of the maxillary alveolar bone, whereas, generally, no correlation exists between bite force and the thickness of the maxillary alveolar bone.

## Figures and Tables

**Table 1:** Demographic and cephalometric variables in the control and AOB groups.

	Control (*n* = 25)	AOB (*n* = 25)	*p*-Value
Sex (male/female)	13/12	9/16	0.393
Age (year)	22.2 ± 4.5	24.2 ± 6.4	0.190
Overbite (mm)	2.7 ± 1.7	−2.1 ± 2.2	<0.001 *
SNA (degree)	82.6 ± 3.1	81.2 ± 3.7	0.168
SNB (degree)	80.8 ± 4.5	79.8 ± 4.2	0.434
ANB (degree)	1.8 ± 3.3	1.4 ± 4.4	0.762
MPA (degree)	26.7 ± 5.9	29.2 ± 6.6	0.160
SN-GoGn (degree)	33.6 ± 5.9	36.6 ± 6.6	0.102
U1 to FH (degree)	120.8 ± 8.8	121.6 ± 7.8	0.710
Bite force (N)	426.7 ± 167.8	363.5 ± 148.8	0.165

Data are shown as the number of subjects and mean ± standard deviation. Abbreviations: AOB, participants with anterior open bite; SNA, angle formed by the sella-nasion (SN) line and the nasion-point A (NA) line; SNB, angle formed by the SN line and the nasion-point B (NB) line; ANB, angle formed by the NA line and the NB line; MPA, angle formed by the Frankfort horizontal (FH) plane and mandibular plane; SN-GoGn, angle formed by the SN line and the gonion (Go)-gnathion (Gn) line; U1 to FH, angle formed by the maxillary incisor axis and FH plane. *: *p* < 0.05.

**Table 2:** Comparison of maxillary morphological variables between the control and AOB groups.

	Control	AOB	*p*-Value
Da (mm)	18.67 ± 2.21	19.76 ± 2.66	0.130
Dp (mm)	14.12 ± 1.35	15.09 ± 1.38	0.016 *
TBa (mm)	1.41 ± 0.17	1.19 ± 0.13	<0.001 *
TPa (mm)	1.44 ± 0.32	1.25 ± 0.16	0.009 *
TBp (mm)	1.44 ± 0.23	1.25 ± 0.24	0.006 *
TPp (mm)	1.36 ± 0.16	1.26 ± 0.20	0.055

Abbreviations: AOB, participants with anterior open bite; Da, distance between the anterior alveolar crest and the palatal plane; Dp, average (i.e., mean of left and right) distance between the posterior alveolar crest and the palatal plane; TBa, thickness of buccal cortical bone of the anterior region; TPa, thickness of palatal cortical bone of the anterior region; TBp, thickness of buccal cortical bone of the posterior region; TPp, thickness of palatal cortical bone of the posterior region. *: *p* < 0.05.

**Table 3:** Coefficients of correlation between the buccal/palatal bone thickness and bite force in the control and AOB groups.

	Coefficients of Correlation (*p*-Value)	
	Control	AOB
TBa vs. bite force	−0.213 (0.295)	−0.036 (0.863)
TPa vs. bite force	−0.227 (0.274)	−0.038 (0.857)
TBp vs. bite force	−0.305 (0.138)	−0.403 (0.046 *)
TPp vs. bite force	−0.159 (0.447)	−0.313 (0.128)

Abbreviations: AOB, participants with anterior open bite; TBa, thickness of buccal cortical bone of the anterior region; TPa, thickness of palatal cortical bone of the anterior region; TBp, thickness of buccal cortical bone of the posterior region; TPp, thickness of palatal cortical bone of the posterior region. *: *p* < 0.05.

## Data Availability

The raw data supporting the conclusions of this article will be made available by the authors on request.
